# Comparative analysis of the main haematological indexes and RNA detection for the diagnosis of SARS-CoV-2 infection

**DOI:** 10.1186/s12879-020-05489-3

**Published:** 2020-10-20

**Authors:** Jialin Xiang, Zuyi Chen, Jie Zhou, Di Tian, Xiusheng Ran, Zhimin Zhang, Shi Shi, Daimin Xiao, Yuanzhong Zhou

**Affiliations:** 1 Department of Laboratory medicine, Affiliated Hospital of Zunyi Medical University, Zunyi, Guizhou 563000 P.R. China; 2 Center for Disease Control and Prevention of Daozhen, Zunyi, Guizhou 563000 P.R. China; 3 Department of Medical Genetics, Zuyi Medical University, Zunyi, Guizhou 563000 P.R. China; 4 Department of Laboratory medicine, The Fourth People’s Hospital of Zunyi, Zunyi, Guizhou 563000 P.R. China; 5 School of Public Health, Zunyi Medcial University, Zunyi, Guizhou 563000 P.R. China

**Keywords:** SARS-CoV-2, Antibody, Real-time PCR, Gold immunochromatography assay, Enzyme-linked immunosorbent assay

## Abstract

**Background:**

Severe acute respiratory syndrome coronavirus 2 (SARS-CoV-2) infection has become a public health emergency of international concern. SARS-CoV-2 RNA detection is the diagnostic criterion for coronavirus disease 2019 (COVID-19). Nevertheless, RNA detection has many limitations, such as being time-consuming and cost-prohibitive, and it must be performed in specialized laboratories. Virus antibody detection is a routine method for screening for multiple viruses, but data about SARS-CoV-2 antibody detection are limited.

**Method:**

Throat swabs and blood were collected from 67 suspected SARS-CoV-2 infection patients at the Affiliated Hospital of Zunyi Medical University and Zunyi Fourth People’s Hospital isolated observation departments. Throat swab samples were subjected to SARS-CoV-2 RNA detection by real-time PCR. Blood was used subjected to SARS-CoV-2 IgG/IgM detection by an enzyme-linked immunosorbent assay (ELISA) and gold immunochromatography assay (GICA). Blood underwent C-reactive protein detection by immunoturbidimetry, and white blood cells, neutrophil percentages and lymphocyte percentages were counted and calculated, respectively. Clinical symptoms, age and lifestyle habits (smoking and drinking) in all patients were recorded. Data were analysed using SPSS version 19. The results were confirmed by T and χ^2^ tests; correlations with detection results were analysed by kappa coefficients. Odds ratio (OR) and corrected OR values were analysed by logistic regression. *P* < 0.05 was considered statistically significant.

**Results:**

Of the 67 patients included in this study, 26 were SARS-CoV-2 RNA-positive. GICA IgM sensitivity was 50.9% (13/26), and specificity was 90.2% (37/41). ELISA IgM sensitivity was 76.9% (20/26), and specificity was 90.2% (37/41). ELISA IgG sensitivity was 76.9% (20/26), and specificity was 95.1% (39/41). The kappa coefficients between RNA detection and ELISA IgG, ELISA IgM, and GICA IgM results were 0.741 (*P* < 0.01), 0.681 (*P <* 0.01) and 0.430 (*P <* 0.01), respectively.

**Conclusion:**

Among the candidate blood indicators, serum IgG and IgM detected by ELISA had the best consistency and validity when compared with standard RNA detection; these indicators can be used as potential preliminary screening tools to identify those who should undergo nucleic acid detection in laboratories without RNA detection abilities or as a supplement to RNA detection.

The first novel coronavirus pneumonia 2019 case was reported in December 2019 [1, 2], and the pathogen responsible for new novel coronavirus pneumonia 2019 was named novel coronavirus 2019 [3, 4]. Novel coronavirus 2019 was found to be highly homologous with severe acute respiratory syndrome coronavirus (SARS-CoV) and Middle East respiratory syndrome coronavirus (MERS-CoV) and had strong transmissibility among human hosts. The World Health Organization (WHO) announced that novel coronavirus 2019 infection was a “public health emergency of international concern” on 31 January 2020 [5–7]. On February 11, 2019, the virus responsible for novel coronavirus 2019 was officially named severe acute respiratory syndrome coronavirus 2 (SAR-CoV-2), and new novel coronavirus pneumonia 2019 was officially named coronavirus disease 2019 (COVID-19) by the WHO. As of April 25, 2020, there were 2,856,339 confirmed SAR-CoV-2-positive cases worldwide. SAR-CoV-2 infection outbreaks have become a serious international public health problem.

SARS-CoV-2 RNA detection is the standard method for SARS-CoV-2 infection diagnosis, and real-time PCR is the main method for SARS-CoV-2 RNA detection; however, COVID-19 imaging results and clinical symptoms have been reported to be inconsistent with the RNA detection results [8]. Several reports noted that some SARS-CoV-2-positive patients had to be tested more than once because of inevitable differences in sampling techniques before the cases were confirmed [9, 10]. SARS-CoV-2 RNA detection is time-consuming and requires specialized working conditions and equipment. Therefore, other faster and easier auxiliary or alternative detection methods are needed for COVID-19 diagnosis. The C-reactive protein (CRP) level, white blood cell count (WBCC), neutrophil percentage (NP) and lymphocyte percentage (LP) are the main auxiliary haematological indexes in COVID-2019 diagnosis [11]. After virus infection, the immune system defends against the virus and produces specific antibodies. Specific IgM antibodies are produced in the early stage after the body is infected; these antibodies can indicate recent infection and disappear rapidly. High-affinity IgG is produced after IgM to elicit long-term immunity. In SARS-infected patient blood, IgM could be detected after 3–6 days and IgG after 8 days of infection [12, 13]. The China National Health and Health Commission issued the Prevention and Control of COVID-19 (Fifth Edition) on February 21, 2020, suggesting that the presence of SARS-CoV-2 antibodies can assist in diagnosing COVID-19.

Currently, a rapid, haematology-based method for the diagnosis of COVID-19 based on blood samples is being explored in clinical applications. Data about SARS-CoV-2 antibody detection specificity and sensitivity are limited, especially data about antibody detection methods, RNA detection methods and main haematological indexes, which are rarely reported. Therefore, in this study, we collected throat swabs and blood samples from suspected COVID-19 patients and tested the for the presence of SARS-CoV-2 RNA and SARS-CoV-2 IgM/IgG antibodies and analysed the main haematological indexes (CRP, WBCC, NP and LP) to evaluate the utility of different indexes in COVID-19 diagnosis and to provide data to assist in COVID-19 prevention.

## Methods

### Design and cases

We enrolled a cohort of patients highly suspected of having COVID-2019 from March 1, 2019, to March 31, 2019, at the Affiliated Hospital of Zunyi Medical University. The patient inclusion criteria were as follows: patients who had close contact with a confirmed COVID-2019 patient and fever as well as computed tomography (CT) features according to the guidelines of diagnosis and treatment of COVID-19 [14, 15]. The exclusion criteria were as follows: confirmed negative or positive patients; and patients with no suspected contact history within 2 weeks. Samples were collected approximately 1 week after close contact with a confirmed COVID-2019 patient or clinical symptoms appeared.

To avoid false negative results, throat swab samples for RNA detection were collected at least twice per patient, with a 24-h interval, and a positive result was considered the final RNA test result if a positive result was obtained. Throat swabs were used for SARS-CoV-2 RNA detection, and blood samples were used for SARS-CoV-2 IgG/IgM detection and main haematological index (CRP, WBCC, NP and LP) evaluation. Clinical symptoms, age and the lifestyle habits of all patients were recorded.

The present study was approved by the Affiliated Hospital of Zunyi Medical University Ethics Committee under approval number KLL-2020-008. Written informed consent was obtained from the patients or their guardians.

### SARS-CoV-2 RNA detection

Throat swabs were collected and transported at 4 °C in RNA storage solution (Zeesan Biotech, Xiamen, Fujian) for SARS-CoV-2 screening. During sample collection, two swabs were obtained from each patient and placed in a single RNA storage solution container for detection. All samples were detected within 6 h of sample collection. SARS-CoV-2 RNA was extracted and purified with a “Virus DNA/RNA Extraction Kit (CDC)” (Xi’an Tianlong Science and Technology, Xi’an, China; batch number: 20021410 T014) according to the manufacturer’s instructions. SARS-CoV-2 RNA was detected by real-time PCR using the “Novel Coronavirus 2019-nCoV RNA Detection Kit” (Daan Gene, Guangzhou, China; batch number: 2020001; limit of detection: 500 copies/mL; test results were compared with 3 different concentrations of positive references and 10 negative references) and “Novel Coronavirus 2019-nCoV RNA Detection Kit” (Shanghai GeneoDX Biotech, Shanghai, China; batch number: COV2020008; limit of detection: 500 copies/mL; test results were compared with 5 different concentrations of positive references and 10 negative references) according to the manufacturers’ instructions. Throat swabs were detected by the “Novel coronavirus 2019-nCoV RNA Detection Kit” (Daan Gene, Guangzhou, China). Suspected positive (cycle threshold near the cut-off threshold of the kit) and positive samples were detected by both real-time PCR kits mentioned above. Negative and positive controls were included in every experiment.

Primers and probes for the two SARS-CoV-2 RNA test kits in this study were established by the Chinese Center for Disease Control and Prevention (CDC), and the two kits are listed in the WHO Emergency Use Listing. The human housekeeping, SARS-CoV-2 ORF1ab and N genes were tested in the same reaction in each sample. If the housekeeping gene was amplified effectively (cut-off value ≤32), the sample and reaction were qualified. When the ORF1ab and N genes in a single sample were amplified effectively (both RNA detection kits used in this study had cut-off values ≤40) at the same time, the sample was considered positive. When only the ORF1ab or N gene in a single sample was amplified effectively, the test was repeated immediately; if the result of the repeated test was consistent with the first test, the sample was considered positive; if not, the sample was considered negative.

### SARS-CoV-2 IgG/IgM detection

Blood serum/plasma samples were used for SARS-CoV-2 IgG/IgM detection. Enzyme-linked immunosorbent assay (ELISA) was used for SARS-CoV-2 IgG and IgM detection with a kit (ZHU HAI LIVZON DIAGNOSTICS INC, Guangdong, China; batch number: 2020020308) according to the manufacturer’s instructions. A gold immunochromatography assay (GICA) was used for SARS-CoV-2 IgM detection and was performed with a kit (Hotgen, Beijing, China; batch number: 20200204) according to the manufacturer’s instructions. Negative and positive controls were included in every experiment.

### CRP, WBC, NP and LP detection

C-reactive protein was detected in blood by immunoturbidimetry (automation equipment: Olympus 5400; Beckman Coulter, California, USA) according to the manufacturer’s instructions. White blood cell counts, neutrophil percentages and lymphocyte percentages were counted and calculated, respectively (automation equipment: Sysmex XN 2000; Sysmex, Kobe, Japan) according to the manufacturer’s instructions.

Data were analysed using SPSS version 19 (IBM, Armonk, NY, USA). The results were confirmed by T and χ^2^ tests; correlations with the detection results were analysed by Kappa coefficients. Odds ratio (OR) and corrected OR values were analysed by logistic regression. *P* < 0.05 was considered statistically significant.

## Results

The mean (+/− SD) age of the patients was 53.82 ± 20.12 years old. Of the 67 patients, 56.7% (39/67) were male, 16.4% (11/67) smoked, and 14.9% (10/67) consumed alcoholic beverages. A total of 55.2% (37/67) of the 67 patients had cough, 17.9% (12/67) had muscle pain, and 65.7% (44/67) had dyspnoea. Twenty-six (38.8%) patients were confirmed to be SARS-CoV-2-positive by RNA detection. The demographics of the SARS-CoV-2-positive and SARS-CoV-2-negative patients are shown in Table 1.

**Table 1:** The demographics and clinical symptoms of the 67 subjects

Variables	NAT(−) (*n* = 41)	NAT(+) (*n* = 26)	Total (*n* = 67)	*P* value
Age (mean ± SD, years)	47.15 ± 18.94	64.35 ± 17.51	53.82 ± 20.12	**0.000**
Sex	n (%)	n (%)	n (%)	
Female	19 (46.3)	10 (38.5)	29 (43.3)	0.526
Male	22 (53.7)	16 (61.5)	38 (56.7)	
Smoking				
Never/former smoker	34 (82.9)	22 (84.6)	56 (83.6)	0.856
Current smoker	7 (17.1)	4 (15.4)	11 (16.4)	
Drinking				
Never/former drinker	37 (90.2)	20 (76.9)	57 (85.1)	0.136
Current drinker	4 (9.8)	6 (23.1)	10 (14.9)	
Cough				
No	25 (61.0)	5 (19.2)	30 (44.8)	**0.001**
Yes	16 (39.0)	21 (80.8)	37 (55.2)	
Muscle aches				
No	32 (78.0)	23 (88.5)	55 (82.1)	0.279
Yes	9 (22.0)	3 (11.5)	12 (17.9)	
Dyspnoea				
No	16 (39.0)	7 (26.9)	23 (34.3)	0.309
Yes	25 (61.0)	19 (73.1)	44 (65.7)	

*SD* standard deviation, *NAT* SARS-CoV-2 RNA, *NAT(−)* SARS-CoV-2 negative, *NAT(+)* SARS-CoV-2 positive. n indicates the patient number

Of the 67 patients, 25.4% (17/67) were GICA IgM positive, 35.8% (24/67) were ELISA IgM positive, and 32.8% (22/67) were ELISA IgG positive. Compared with the reference ranges of healthy people, 53.7% (36/67) of patients had higher levels of plasma CRP, 53.7% (36/67) of patients had higher white blood cell counts, 49.3% (33/67) of patients had higher neutrophil percentages, and 17.9% (12/67) of patients had higher lymphocyte percentages. The GICA IgM, ELISA IgM, ELISA IgG, CRP, WBCC, NP and LP results in SARS-CoV-2-positive as well as SARS-CoV-2-negative patients are shown in Table 2. Only the GICA IgM (*P* < 0.01), ELISA IgM (*P <* 0.01), and ELISA IgG (*P <* 0.01) results between SARS-CoV-2 positive and negative patients were statistically significant. Details are shown in Table 2.

**Table 2:** The haematological test results of the 67 subjects

Variables	NAT(−)	NAT(+)	Total n (%)	*P* value
Gold immunochromatography assay (IgM)				
Negative	37 (90.2)	13 (50.0)	50 (74.6)	**0.000**
Positive	4 (9.8)	13 (50.0)	17 (25.4)	
Enzyme-linked immunosorbent assay (IgM)				
Negative	37 (90.2)	6 (23.1)	43 (64.2)	**0.000**
Positive	4 (9.8)	20 (76.9)	24 (35.8)	
Enzyme-linked immunosorbent assay (IgG)				
Negative	39 (95.1)	6 (23.1)	45 (67.2)	**0.000**
Positive	2 (4.9)	20 (76.9)	22 (32.8)	
C-reactive protein				
Normal	20 (48.8)	11 (42.3)	31 (46.3)	0.569
Elevated	21 (51.2)	15 (57.7)	36 (53.7)	
White blood cell count				
Normal	27 (65.9)	20 (76.9)	47 (70.1)	0.335
Elevated	14 (34.1)	6 (23.1)	20 (29.9)	
Neutrophil percentage				
Normal	24 (58.5)	10 (38.5)	34 (50.7)	0.109
Elevated	17 (41.5)	16 (61.5)	33 (49.3)	
Lymphocyte percentage				
Normal	31 (75.6)	24 (92.3)	55 (82.1)	0.082
Elevated	10 (24.4)	2 (7.7)	12 (17.9)	

*NAT* SARS-CoV-2 RNA, *NAT(−)* SARS-CoV-2 negative, *NAT(+)* SARS-CoV-2 positive. n indicates the patient number. GICA positive and negative predictive values were 13/17 (76.5%) and 37/50 (74.0%), respectively. ELISA IgM positive and negative predictive values were 20/24 (83.3%) and 37/43 (86.0%), respectively. ELISA IgG positive and negative predictive values were 20/22 (90.9%) and 39/45 (86.7%), respectively

Based on the RNA detection results, GICA IgM sensitivity was 50.0% (13/26), and specificity was 90.2% (37/41). ELISA IgM sensitivity was 76.9% (20/26), specificity was 90.2% (37/41). ELISA IgG sensitivity was 76.9% (20/26), and specificity was 95.1% (39/41). Details are shown in Table 2.

The GICA positive and negative predictive values were 76.5% (13/17) and 74.0% (37/50), respectively. The ELISA IgM positive and negative predictive values were 83.3% (20/24) and 86.0% (37/43), respectively. The ELISA IgG positive and negative predictive values were 90.9% (20/22) and 86.7% (39/45), respectively (calculated from Table 2).

The kappa coefficients between SARS-CoV-2 RNA and GICA IgM, ELISA IgM and ELISA IgG detection were 0.741 (*P* < 0.01), 0.681 (*P <* 0.01) and 0.430 (*P <* 0.01), respectively. The kappa coefficients between SARS-CoV-2 RNA detection and CRP (*P* > 0.05), WBCC (*P >* 0.05), NP (*P >* 0.05) and LP (*P >* 0.05) were not consistent. Details are shown in Table 3.

**Table 3:** Kappa coefficients between detection results

	NAT(+)	GICA(+) (IgM)	ELISA(+) (IgM)	ELISA(+) (IgG)	CRP(+)	WBCC(+)	NP(+)	LP(+)
NAT(+)	1.000	0.430^**^	0.681^**^	0.741^**^	0.06	−0.116	0.191	−0.185
GICA(+) (IgM)	–	1.000	0.480^**^	0.461	−0.065	−0.006	0.038	−0.266^*^
ELISA(+) (IgM)	–	–	1.000	0.868^**^	0.065	−0.078	0.251^*^	−0.241^*^
ELISA(+) (IgG)	–	–	–	1.000	0.069	−0.039	0.310^**^	−0.255^*^
CRP(+)	–	–	–	–	1.000	0.304^**^	0.374^**^	−0.082
WBCC(+)	–	–	–	–	–	1.000	0.309^**^	−0.034
NP(+)	–	–	–	–	–	–	1.000	0.066
LP(+)	–	–	–	–	–	–	–	1.000

^**^ *P <* 0.01, ^*^ *P* < 0.05

The odds ratio of SARS-CoV-2 RNA positivity in GICA IgM-positive individuals was 9.25 compared with that in GICA IgM-negative individuals (correction value 29.79, *P* = 0.01). The odds ratio of SARS-CoV-2 RNA positivity in ELISA IgM-positive individuals was 30.83 compared with that in ELISA IgM-negative individuals (correction value 27.09, *P* < 0.01). The odds ratio of SARS-CoV-2 RNA positivity in ELISA IgG-positive individuals was 65.00 compared with that in ELISA IgG-negative individuals (correction value 84.16, *P <* 0.01). The odds ratios of SARS-CoV-2 RNA positivity in those with elevated CRP (*P* > 0.05), WBCCs (*P >* 0.05), NPs (*P >* 0.05), LPs (*P >* 0.05) compared with the odds rations in those without elevated values were not consistent. Details are shown in Table 4.

**Table 4:** Odds ratios for abnormal laboratory results according to the RNA testing results

	Model 1^a^		Model 2^b^	
	OR (95% CI)	*p* –value	OR (95% CI)	*p* –value
GICA IgM(+)	9.25 (2.55, 33.49)	**0.001**	29.79 (4.20, 211.34)	**0.001**
ELISA IgM(+)	30.83 (7.78, 122.21)	**0.000**	27.09 (4.87, 150.82)	**0.000**
ELISA IgG(+)	65.00 (12.01, 351.79)	**0.000**	84.16 (7.51, 943.41)	**0.000**
CRP(+)	1.30 (0.48, 3.50)	0.605	1.08 (0.16, 7.51)	0.935
WBCC(+)	0.58 (0.19, 1.77)	0.337	0.54 (0.08, 3.60)	0.528
NP(+)	2.26 (0.83, 6.17)	0.112	5.29 (0.77, 36.52)	0.091
LP(+)	0.26 (0.05, 1.29)	0.099	1.21 (0.04, 5.25)	0.527

A logistic regression model was used for analysis. Different blood test results were used as dependent variables, and different RNA test results (classified variables) were used as independent variables

^a^means not adjusted for covariates

^b^means adjusted for covariates such as age and cough

*CI* confidence interval, + positive

## Discussion

In this study, based on SARS-CoV-2 RNA test results, there were significant differences in the IgM and IgG antibody detection results between SARS-CoV-2-positive and SARS-CoV-2-negative patients by ELISA (*P* < 0.01) and GICA (*P <* 0.01) (see Table 2). SARS-CoV-2 IgM and IgG antibody detection methods had high specificity; and ELISA IgG had the highest specificity (95.1%, 39/41) and the highest sensitivity (90.2%, 37/41). The positive predictive values (90.9%, 20/22) and negative predictive values (86.7%, 39/45) of the ELISA IgM and IgG methods were the highest, while the positive predictive value (83.3%, 20/24) and negative predictive value (86.0%, 37/43) of GICA were lower than those obtained by ELISA. Both the ELISA IgG and IgM methods had high sensitivity (76.9%, 20/26), while the sensitivity of GICA was low (50.9%, 13/26). CRP (*P* > 0.05), WBCC (*P >* 0.05), NP (*P >* 0.05) and LP (*P >* 0.05) were not specific.

SARS-CoV-2 is transmitted by respiratory droplets and close contact; it is easily transmitted in all populations [16]. Generally, fever, fatigue and cough are the main manifestations of COVID-19; a small number of patients have symptoms of nasal obstruction, runny nose, and diarrhoea, while some severe patients have obvious dyspnoea [16–18]. In this study, we found that sex, smoking status, and drinking status had no significant association with SARS-CoV-2 infection, but age had a significant association with SARS-CoV-2 infection (shown in Table 1). Although all populations are easily infected by SARS-CoV-2 [16], older people are more likely to be infected. Dyspnoea and muscle pain were not significantly associated with SARS-CoV-2 infection, but cough was significantly associated with SARS-CoV-2 infection (shown in Table 1); cough may be another important early clinical symptom predictive of COVID-19 diagnosis [16]. CRP, the WBCC, the NP and the LP had no significant associations with SARS-CoV-2 infection (all *P* > 0.05). Fever and CT features were sample screening criteria [16, 18], so fever and CT features were not analysed.

Real-time PCR is the most common detection method for SARS-CoV-2 diagnosis [19]. Nevertheless, SARS-CoV-2 RNA real-time PCR must be performed in a specific experimental environment (at least a bio-safety level 2 laboratory) that requires specialized experimental equipment and skilled personnel. The SARS-CoV-2 RNA real-time PCR detection method (including RNA extraction) generally requires 3–5 h [18]. The SARS-CoV-2 antibody ELISA generally requires 2–3 h and does not require a specific experimental environment [17]. The SARS-CoV-2 antibody GICA requires approximately 20 min and does not require a specific experimental environment [17].

In this study, based on the SARS-CoV-2 RNA test results, the ORs for SARS-CoV-2 RNA positivity in IgM−/IgG-positive and negative patients were calculated. The confirmed OR in the ELISA IgG-positive group compared with that in IgG-negative group was the highest (OR = 65.00, corrected OR = 81.64, *P* < 0.01), followed by those in ELISA IgM-positive (OR = 30.83, corrected OR = 27.09, *P* < 0.01) and GICA IgM-positive groups (OR = 9.25, corrected OR = 29.79, *P* < 0.01). ELISA IgM detection was the most sensitive method in preliminary screening of a high-risk population in this study; CRP (*P* > 0.05), WBCCs (*P >* 0.05), NPs (*P >* 0.05) and LPs (*P* > 0.05) could not accurately identify high-risk patients. The kappa coefficients between RNA detection and ELISA IgG, ELISA IgM, and GICA IgM detection were 0.741 (*P <* 0.01), 0.681 (*P <* 0.01) and 0.430 (*P <* 0.01), respectively. The consistency between ELISA IgG detection and RNA detection was good, but the consistency between GICA IgM detection and RNA detection was unsatisfactory. The kappa coefficients between RNA detection and the CRP level (*P* > 0.05), WBCC (*P >* 0.05), NP (*P >* 0.05), and LP (*P >* 0.05) were not statistically significant.

Serum samples for antibody detection were easier to collect (lower risk during sample collection) and preserve (the antibody is more stable than RNA) than throat swabs. The ELISA and GICA methods were easier, cheaper and faster than real-time PCR and may be more suitable for primary hospitals. Antibody detection cannot completely replace RNA detection, but it can be auxiliary to RNA detection [19]. ELISA IgG was the best antibody detection method in this study.

There were some limitation in this study. The sample size was small, and it was difficult to ascertain the sample collection time point in early screening. In the future, more clinical manifestations and detection indicators should be compared. Additional studies should be performed to validate the accuracy of SARS-CoV-2 antibody detection.

## Conclusions

Among the candidate blood indicators, the serum levels of IgG and IgM detected by ELISA had the best consistency and validity when compared with standard RNA detection; antibody detection can be used as a potential preliminary screening method to identify those who should undergo nucleic acid detection in laboratories without RNA detection abilities or as a supplement to RNA detection.

## Data Availability

The datasets used and/or analysed during the current study are available from the corresponding author on reasonable request.

## Abbreviations

SARS-CoV-2: Severe acute respiratory syndrome coronavirus 2
SARS: Severe acute respiratory syndrome coronavirus
MERS: Middle East respiratory syndrome coronavirus
COVID-19: Coronavirus disease 2019
ELISA: Enzyme-linked immunosorbent assay
GICA: Gold immunochromatography assay
IgM: Immunoglobulin M
IgG: Immunoglobulin G
CRP: C-reactive protein
WBCC: White blood cell count
NP: Neutrophil percentage
LP: Lymphocyte percentage
CT: Computed tomography
CDC: Centers for disease control and prevention
OR: Odds ratio
